# Supplementary material for: HIVprotI: an integrated web based platform for prediction and design of HIV proteins inhibitors
Source: J Cheminform. 2018 Mar 9;10:12. doi: 10.1186/s13321-018-0266-y (PMC5845081; doi:10.1186/s13321-018-0266-y)
Supplement: Supplementary file 2 — Additional file 2. Source code of HIVProtI web server. [file 13321_2018_266_MOESM2_ESM.zip › HIVprotI_Source-code/hivprotI/weka-3-6-6/doc/weka/clusterers/HierarchicalClusterer.html]

HierarchicalClusterer


---


|  |  |  |
| --- | --- | --- |
| **Overview** \| **Package** \| **Class** \| **Tree** \| **Deprecated** \| **Index** \| **Help** \| **Weka's home** | |  |
| **PREV CLASS**   **NEXT CLASS** | **FRAMES**    **NO FRAMES**     **All Classes** |  |
| SUMMARY: NESTED \| FIELD \| CONSTR \| METHOD | DETAIL: FIELD \| CONSTR \| METHOD |  |


---


## weka.clusterers Class HierarchicalClusterer

```
java.lang.Object
  weka.clusterers.AbstractClusterer
      weka.clusterers.HierarchicalClusterer
```

**All Implemented Interfaces:**: java.io.Serializable, java.lang.Cloneable, Clusterer, CapabilitiesHandler, Drawable, OptionHandler, RevisionHandler

---

``` public class HierarchicalClusterer extends AbstractClusterer implements OptionHandler, CapabilitiesHandler, Drawable ```

Hierarchical clustering class.
Implements a number of classic hierarchical clustering methods.

Valid options are:

```
 -N
  number of clusters
```

```
 -L
  Link type (Single, Complete, Average, Mean, Centroid, Ward, Adjusted complete, Neighbor Joining)
  [SINGLE|COMPLETE|AVERAGE|MEAN|CENTROID|WARD|ADJCOMLPETE|NEIGHBOR_JOINING]
```

```
 -A
 Distance function to use. (default: weka.core.EuclideanDistance)
```

```
 -P
 Print hierarchy in Newick format, which can be used for display in other programs.
```

```
 -D
 If set, classifier is run in debug mode and may output additional info to the console.
```

```
 -B
 \If set, distance is interpreted as branch length, otherwise it is node height.
```

**Version:**
:   $Revision: 6592 $

**Author:**
:   Remco Bouckaert (rrb@xm.co.nz, remco@cs.waikato.ac.nz), Eibe Frank (eibe@cs.waikato.ac.nz)

**See Also:**: Serialized Form

---

| **Field Summary** | |
| --- | --- |
| `static Tag[]` | `TAGS_LINK_TYPE` |

**Fields inherited from interface weka.core.Drawable**

`BayesNet, Newick, NOT_DRAWABLE, TREE`


**Constructor Summary**

`HierarchicalClusterer()`


| **Method Summary** | |
| --- | --- |
| `void` | `buildClusterer(Instances data)`             Generates a clusterer. |
| `int` | `clusterInstance(Instance instance)`             Classifies a given instance. |
| `java.lang.String` | `debugTipText()`             Returns the tip text for this property |
| `java.lang.String` | `distanceFunctionTipText()` |
| `java.lang.String` | `distanceIsBranchLengthTipText()` |
| `double[]` | `distributionForInstance(Instance instance)`             Predicts the cluster memberships for a given instance. |
| `Capabilities` | `getCapabilities()`             Returns the Capabilities of this clusterer. |
| `boolean` | `getDebug()`             Get whether debugging is turned on. |
| `DistanceFunction` | `getDistanceFunction()` |
| `boolean` | `getDistanceIsBranchLength()` |
| `SelectedTag` | `getLinkType()` |
| `int` | `getNumClusters()` |
| `java.lang.String[]` | `getOptions()`             Gets the current settings of the clusterer. |
| `boolean` | `getPrintNewick()` |
| `java.lang.String` | `getRevision()`             Returns the revision string. |
| `java.lang.String` | `globalInfo()`             This will return a string describing the clusterer. |
| `java.lang.String` | `graph()`             Returns a string that describes a graph representing the object. |
| `int` | `graphType()`             Returns the type of graph representing the object. |
| `java.lang.String` | `linkTypeTipText()` |
| `java.util.Enumeration` | `listOptions()`             Returns an enumeration describing the available options. |
| `static void` | `main(java.lang.String[] argv)` |
| `int` | `numberOfClusters()`             Returns the number of clusters. |
| `java.lang.String` | `numClustersTipText()` |
| `java.lang.String` | `printNewickTipText()` |
| `void` | `setDebug(boolean debug)`             Set debugging mode. |
| `void` | `setDistanceFunction(DistanceFunction distanceFunction)` |
| `void` | `setDistanceIsBranchLength(boolean bDistanceIsHeight)` |
| `void` | `setLinkType(SelectedTag newLinkType)` |
| `void` | `setNumClusters(int nClusters)` |
| `void` | `setOptions(java.lang.String[] options)`             Parses a given list of options. |
| `void` | `setPrintNewick(boolean bPrintNewick)` |
| `java.lang.String` | `toString()` |

**Methods inherited from class weka.clusterers.AbstractClusterer**

`forName, makeCopies, makeCopy`

**Methods inherited from class java.lang.Object**

`equals, getClass, hashCode, notify, notifyAll, wait, wait, wait`

**Field Detail**

### TAGS\_LINK\_TYPE

```
public static final Tag[] TAGS_LINK_TYPE
```


**Constructor Detail**

### HierarchicalClusterer

```
public HierarchicalClusterer()
```


**Method Detail**

### setNumClusters

```
public void setNumClusters(int nClusters)
```

---


### getNumClusters

```
public int getNumClusters()
```

---


### getDistanceFunction

```
public DistanceFunction getDistanceFunction()
```

---


### setDistanceFunction

```
public void setDistanceFunction(DistanceFunction distanceFunction)
```

---


### getPrintNewick

```
public boolean getPrintNewick()
```

---


### setPrintNewick

```
public void setPrintNewick(boolean bPrintNewick)
```

---


### setLinkType

```
public void setLinkType(SelectedTag newLinkType)
```

---


### getLinkType

```
public SelectedTag getLinkType()
```

---


### buildClusterer

```
public void buildClusterer(Instances data)
                    throws java.lang.Exception
```

:   **Description copied from class: `AbstractClusterer`**
:   Generates a clusterer. Has to initialize all fields of the clusterer
    that are not being set via options.

    :   **Specified by:**: `buildClusterer` in interface `Clusterer` **Specified by:**: `buildClusterer` in class `AbstractClusterer`
    :   **Parameters:**: `data` - set of instances serving as training data **Throws:**: `java.lang.Exception` - if the clusterer has not been generated successfully

---


### clusterInstance

```
public int clusterInstance(Instance instance)
                    throws java.lang.Exception
```

:   **Description copied from class: `AbstractClusterer`**
:   Classifies a given instance. Either this or distributionForInstance()
    needs to be implemented by subclasses.

    :   **Specified by:**: `clusterInstance` in interface `Clusterer` **Overrides:**: `clusterInstance` in class `AbstractClusterer`
    :   **Parameters:**: `instance` - the instance to be assigned to a cluster **Returns:**: the number of the assigned cluster as an integer **Throws:**: `java.lang.Exception` - if instance could not be clustered successfully

---


### distributionForInstance

```
public double[] distributionForInstance(Instance instance)
                                 throws java.lang.Exception
```

:   **Description copied from class: `AbstractClusterer`**
:   Predicts the cluster memberships for a given instance. Either
    this or clusterInstance() needs to be implemented by subclasses.

    :   **Specified by:**: `distributionForInstance` in interface `Clusterer` **Overrides:**: `distributionForInstance` in class `AbstractClusterer`
    :   **Parameters:**: `instance` - the instance to be assigned a cluster. **Returns:**: an array containing the estimated membership probabilities of the test instance in each cluster (this should sum to at most 1) **Throws:**: `java.lang.Exception` - if distribution could not be computed successfully

---


### getCapabilities

```
public Capabilities getCapabilities()
```

:   **Description copied from class: `AbstractClusterer`**
:   Returns the Capabilities of this clusterer. Derived classifiers have to
    override this method to enable capabilities.

    :   **Specified by:**: `getCapabilities` in interface `Clusterer` **Specified by:**: `getCapabilities` in interface `CapabilitiesHandler` **Overrides:**: `getCapabilities` in class `AbstractClusterer`
    :   **Returns:**: the capabilities of this object **See Also:**: `Capabilities`

---


### numberOfClusters

```
public int numberOfClusters()
                     throws java.lang.Exception
```

:   **Description copied from class: `AbstractClusterer`**
:   Returns the number of clusters.

    :   **Specified by:**: `numberOfClusters` in interface `Clusterer` **Specified by:**: `numberOfClusters` in class `AbstractClusterer`
    :   **Returns:**: the number of clusters generated for a training dataset. **Throws:**: `java.lang.Exception` - if number of clusters could not be returned successfully

---


### listOptions

```
public java.util.Enumeration listOptions()
```

:   Returns an enumeration describing the available options.

    :   **Specified by:**: `listOptions` in interface `OptionHandler`
    :   **Returns:**: an enumeration of all the available options.

---


### setOptions

```
public void setOptions(java.lang.String[] options)
                throws java.lang.Exception
```

:   Parses a given list of options.


    Valid options are:

    :   **Specified by:**: `setOptions` in interface `OptionHandler`
    :   **Parameters:**: `options` - the list of options as an array of strings **Throws:**: `java.lang.Exception` - if an option is not supported

---


### getOptions

```
public java.lang.String[] getOptions()
```

:   Gets the current settings of the clusterer.

    :   **Specified by:**: `getOptions` in interface `OptionHandler`
    :   **Returns:**: an array of strings suitable for passing to setOptions()

---


### toString

```
public java.lang.String toString()
```

:   **Overrides:**: `toString` in class `java.lang.Object`

---


### setDebug

```
public void setDebug(boolean debug)
```

:   Set debugging mode.

    :   **Parameters:**: `debug` - true if debug output should be printed

---


### getDebug

```
public boolean getDebug()
```

:   Get whether debugging is turned on.

    :   **Returns:**: true if debugging output is on

---


### getDistanceIsBranchLength

```
public boolean getDistanceIsBranchLength()
```

---


### setDistanceIsBranchLength

```
public void setDistanceIsBranchLength(boolean bDistanceIsHeight)
```

---


### distanceIsBranchLengthTipText

```
public java.lang.String distanceIsBranchLengthTipText()
```

---


### debugTipText

```
public java.lang.String debugTipText()
```

:   Returns the tip text for this property

    :   **Returns:**: tip text for this property suitable for displaying in the explorer/experimenter gui

---


### numClustersTipText

```
public java.lang.String numClustersTipText()
```

:   **Returns:**: a string to describe the NumClusters

---


### printNewickTipText

```
public java.lang.String printNewickTipText()
```

:   **Returns:**: a string to describe the print Newick flag

---


### distanceFunctionTipText

```
public java.lang.String distanceFunctionTipText()
```

:   **Returns:**: a string to describe the distance function

---


### linkTypeTipText

```
public java.lang.String linkTypeTipText()
```

:   **Returns:**: a string to describe the Link type

---


### globalInfo

```
public java.lang.String globalInfo()
```

:   This will return a string describing the clusterer.

    :   **Returns:**: The string.

---


### main

```
public static void main(java.lang.String[] argv)
```

---


### graph

```
public java.lang.String graph()
                       throws java.lang.Exception
```

:   **Description copied from interface: `Drawable`**
:   Returns a string that describes a graph representing
    the object. The string should be in XMLBIF ver.
    0.3 format if the graph is a BayesNet, otherwise
    it should be in dotty format.

    :   **Specified by:**: `graph` in interface `Drawable`
    :   **Returns:**: the graph described by a string **Throws:**: `java.lang.Exception` - if the graph can't be computed

---


### graphType

```
public int graphType()
```

:   **Description copied from interface: `Drawable`**
:   Returns the type of graph representing
    the object.

    :   **Specified by:**: `graphType` in interface `Drawable`
    :   **Returns:**: the type of graph representing the object

---


### getRevision

```
public java.lang.String getRevision()
```

:   Returns the revision string.

    :   **Specified by:**: `getRevision` in interface `RevisionHandler` **Overrides:**: `getRevision` in class `AbstractClusterer`
    :   **Returns:**: the revision


---


|  |  |  |
| --- | --- | --- |
| **Overview** \| **Package** \| **Class** \| **Tree** \| **Deprecated** \| **Index** \| **Help** \| **Weka's home** | |  |
| **PREV CLASS**   **NEXT CLASS** | **FRAMES**    **NO FRAMES**     **All Classes** |  |
| SUMMARY: NESTED \| FIELD \| CONSTR \| METHOD | DETAIL: FIELD \| CONSTR \| METHOD |  |


---
